# The association between self-rated health and social environments, health behaviors and health outcomes: a structural equation analysis

**DOI:** 10.1186/s12889-018-5323-y

**Published:** 2018-04-03

**Authors:** Bevan Adrian Craig, Darren Peter Morton, Peter John Morey, Lillian Marton Kent, Alva Barry Gane, Terry Leslie Butler, Paul Meredith Rankin, Kevin Ross Price

**Affiliations:** 1 Lifestyle Research Centre, Avondale College of Higher Education, Cooranbong, NSW Australia; 2 Faculty of Education, Business and Science, Avondale College of Higher Education, Cooranbong, NSW Australia; 3 Avondale College of Higher Education, Cooranbong, NSW Australia; 4 Loma Linda University, California, USA; 5 Adventist Health Seventh-Day Adventist Church, Wahroonga, NSW Australia

**Keywords:** Self-rated health, Adolescent health behaviors, Mental health, Family dynamics, Adverse childhood experiences, Social determinants of health

## Abstract

**Background:**

The factors shaping the health of the current generation of adolescents are multi-dimensional and complex. The purpose of this study was to explore the determinants of self-rated health (SRH) of adolescents attending a faith-based school system in Australia.

**Methods:**

A total of 788 students attending 21 Seventh-day Adventist schools in Australia responded to a health and lifestyle survey that assessed SRH as well as potential determinants of SRH including the health outcomes mental health, vitality, body mass index (BMI), select health behaviors, social factors and personal demographics. Structural equation modeling was used to analyze the data and examine the direct and indirect effects of these factors on SRH.

**Results:**

The structural model developed was a good fit with the data. The health outcome mental health had the strongest association with SRH (β = 0.17). Several upstream variables were also associated with higher SRH ratings. The health behavior sleep hours had the strongest association with SRH (β_total_ = 0.178) followed by fruit/vegetable consumption (β_total_ = 0.144), physical activity (β_total_ = 0.135) and a vegetarian diet (β_total_ = 0.103). Of the demographic and social variables measured, adverse childhood experiences (ACEs) had the strongest association with SRH (β_total_ = − 0.125), negatively influencing SRH, and gender also associated with an increase in SRH (β_total_ = 0.092), with the influence of these factors being mediated through other variables in the model.

**Conclusions:**

This study presents a conceptual model that illustrates the complex network of factors concomitantly associated with SRH in adolescents. The outcomes of the study provide insights into the determinants of adolescent SRH which may inform priority areas for improving this construct.

## Background

The factors shaping the health of the current and largest generation of adolescents in human history are multi-dimensional, complex and unparalleled [1, 2]. Until recently, adolescent health has been overlooked and misunderstood, which is one reason why adolescents historically have had fewer health gains than any other age group [1], and hence are now central to a number of major current global health challenges [2]. However, addressing adolescent health potentially provides a triple dividend with benefits now, later in adult life and for the next generation of children [1]. Further, the period of adolescence may also provide a second chance to reduce or reverse early-life disadvantage [3].

In recent decades, theorists have argued that understanding the factors driving growing adolescent health concerns requires a broad focus [4]. Clearly, risk and protective factors of adolescent health include levels of physical activity, substance use, alcohol consumption, tobacco usage [5], diet [1], adolescent abnormal weight (underweight, over weight) and mental health [5]. However, it has been asserted that as well as focusing on an individual’s health risk and protective factors, the upstream social patterns and structures in which adolescents exist needs to be considered [4]. Ecological theorists [6] argue that an individual’s social environment, both present and past, influence their health behaviors and health outcomes, mediated by other factors including their demographics and physical and psychological makeup.

**Fig. 1:**
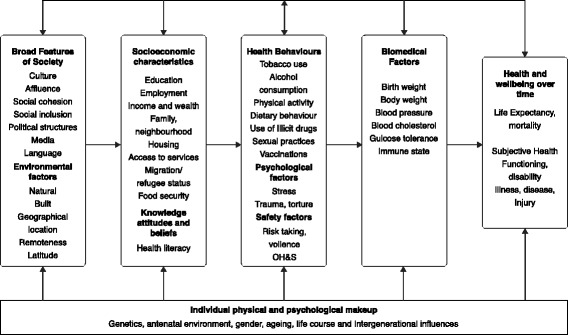
Conceptual Framework for Determinants of Health

Social environments are multifaceted and include peer, school, community, societal, cultural, new media influences and family dynamics [7]. Adverse childhood experiences (ACEs) such as psychological, physical or sexual abuse, violence, parental substance abuse, parental separation/divorce, parental incarceration or death of a parent, close relative or friend may also influence health behaviors and health outcomes in adolescents [8–11]. Conceptual frameworks have been developed to represent the complex web of causal “pathways” through which social factors interact with an individual’s health risk and protective factors throughout the life course (Fig. 1) [12]. However, these models have not been tested among adolescent populations.

Self-rated heath is a legitimate and stable construct used in adolescent populations [13–21]. Reviews by Idler and Benyamini [19] proposed that an individual’s health status cannot be assessed without the SRH measure as it captures an “irreplaceable dimension of health status,” spanning past, present and future physical, behavioral, emotional, cognitive [22] and social [20] dimensions of health.

Widespread agreement in the literature [15, 23–25] recognizes that SRH is a complex parameter affected by multifarious determinants. Specifically, SHR is influenced by higher body mass index [17], mental health (emotional wellbeing, acceptance [20], self-esteem [16]) select health behaviors [20] (diet [18], physical activity [20] substance abuse [16], lack of sleep [14]), demographics (age, gender [13]) and social factors (family dynamics, child-parent relationships, school achievement [16], positive school experiences [13], socio-economic status [18], religion [26]). Many of these factors have complex interrelationships [23], directly or indirectly affecting self-perception of health status [15].

While an increasing number of studies have been reported on SRH among adolescents [13], most research in this field [13–18, 24–26] address only select factors affecting health status, and thus yield only partial or confounded information on the determinants of adolescent health [23]. Investigations need to assess concomitantly the factors associated with this multi-faceted health measure [23].

Utilizing structural equation modeling and SRH as a measure of health status, this study aimed to explore concomitantly the complex relationships between SRH and social environments, health behaviors and health outcomes among adolescents attending a faith-based school system in Australia.

## Methods

### Study design and participants

In 2012, 1734 students aged 12 to 18 years of age responded to a health and lifestyle survey that was administered in 21 Seventh-day Adventist (Adventist) private secondary schools in Australia. The database created by this survey has been used in previous studies [27, 28]. Seven hundred and eighty eight students from this database met the inclusion criteria for this study which included useable data for the following domains: SRH, BMI, Mental Health, and Vitality. Notably, BMI data were not collected on over 900 students in the database, hence these cases did not meet the inclusion criteria.

The study was approved by the Avondale College of Higher Education Human Research Ethics Committee (No:2011:21), and participation in the study was voluntary and anonymous.

**Fig. 2:**
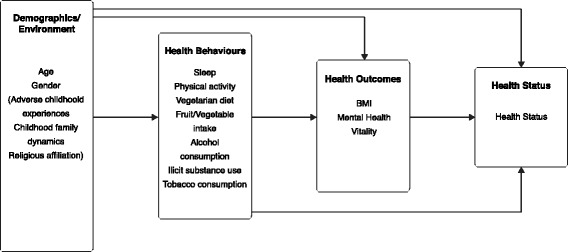
Hypothesized Model for Factors Associated with Self-Rated Health in Adolescents

A hypothesized model informed by ecological theory and the conceptual framework for determinants of health [12] is presented in Fig. 2. The dependent variable was the measure SRH. In order to concomitantly explore factors associated with SRH yet retain a parsimonious model, we delimited the study by restricting the explanatory variables to the following: health outcome variables (BMI, mental health, vitality); health behavior variables (sleep hours per night, amount of moderate to vigorous physical activity, fruit and vegetable intake, vegetarian diet, marijuana use, alcohol consumption and tobacco use); and demographic and social variables (age, gender, ACEs, Childhood family dynamics (CFD), religious affiliation).

### Survey instrument

The survey instrument recorded the participant’s SRH as well as: BMI; measures of mental health and vitality; selected health behaviors; personal demographics; and social influences.

#### Self-rated health status

SRH status was assessed with a single item involving a five-point Likert scale ranging from “Excellent,” “Very Good,” “Good,” “Fair” and “Poor.”

#### Body mass index

Height and weight were self-reported and used to calculate BMI using the standard equation: BMI = weight in kg/(height in m)^2^.

#### Mental health and vitality

Mental health and vitality were measured using the validated and reliable [29] five-item mental health and four-item vitality subscales from the SF-36 [30]. These subscales measure general mental health status and assess the individual’s energy and fatigue. Each item in the mental health and vitality subscales has six response options ranging from “All of the time” to “Not at all.” Standardized scores for these subscales were calculated creating a 0-100 scale according to the standard procedure for calculating the mental health and vitality scores [31]. Higher scores indicated better mental health and vitality. Internal reliability of the mental health and vitality subscales have been reported at α = .78 to .87 and α = .72 to .87 respectively in studies across eleven countries [32]. As seen in Table 1, the reliability of vitality in this study was comparatively lower than in these reports.

**Table 1:** Descriptive statistics and scale reliability of variables used in the analysis

Variables	N	%	Mean	Standard Deviation	Min	Max	Scale Reliability (α)
Age	788		14.90	± 1.57	12	18	
Gender							
Males Females	379 409	48.1% 51.9%					
ACEs score	788		1.39	± 1.60	0	9	
Childhood family dynamics scale	788		23.93	± 4.95	0	30	.83
Religious affiliation							
Adventist	383	48.6%					
Not Adventist	405	51.4%					
Sleep hours per night	788		7.92	± 1.30	3	10	
30 min sessions of MVPA per week	788		3.69	± 1.72	0	6	
Serves of fruit and vegetables per day	788		5.31	± 2.39	0	12	
Vegetarian diet							
Yes No	123 665	15.6% 84.4%					
Drinks of alcohol in the last four weeks	788		1.46	± 6.48	0	20	
Cigarettes smoked in the last four weeks	788		0.84	± 5.84	0	30	
Times smoked Marijuana in the last four weeks	788		0.32	± 2.78	0	5	
BMI	788		20.25	± 2.89	14.30	30.07	
Mental health scale	788		65.73	± 18.00	0	100	.77
Vitality scale	788		57.32	± 17.73	0	100	.66
Self-rated health							
Poor Fair Good Very Good Excellent	6 42 258 341 141	0.6% 5.2% 32.8% 43.4% 17.9%					

*Abbreviation: α* cronbach’s alpha

#### Selected health behaviors

Sleep hygiene was assessed by an item that asked: “How many hours do you usually sleep per night?”, with eight response options ranging from “3 h or less” to “10 h or more”. Physical activity was measured by an item that asked: “How many times per week do you usually do any vigorous or moderate physical activity for at least 30 minutes?”, with seven response options ranging from “none” to “6 or more times” [33]. Fruit and vegetable intake was assessed using food frequency questions adapted from items previously used in adolescent studies [34]. Fruit consumption was measured by an item that asked: “How many serves of fruit do you usually eat each day? (A serve = 1 medium piece or 2 small pieces of fruit or 1 cup of diced pieces)”. Response options ranged from “I do not eat fruit” to “6 serves or more”. Vegetable consumption was measured by an item that asked: “How many serves of vegetables and salad vegetables (exclude potatoes) do you usually eat each day? (A serve = 1/2 cup of cooked vegetables or 1 cup of salad vegetables)”. Response options ranged from “I do not eat vegetables” to “6 servers or more”. The fruit and vegetable items were summed to provide an overall fruit and vegetable intake score. As a measure of the respondents’ overall diet, an item asked: “How would you describe your usual diet?” Response options included: 1. “Total Vegetarian (no animal products: no red meat, chicken, fish, eggs, or milk/dairy products)”; 2. “Lacto–ovo vegetarian (no red meat, chicken or fish but diet includes eggs and/or milk/dairy products)”; 3. “Pesco–vegetarian (diet includes fish but no red meat or chicken, but may include eggs and/or milk/dairy products)”; 4. “Non-Vegetarian (diet includes red meat, chicken, fish)”. For the purpose of this study, this item was dichotomized as a vegetarian (response 1 or 2) or non-vegetarian diet (response 3 or 4). This item was included in the study because a high proportion of Adventists adhere to a vegetarian diet [35]. Alcohol consumption, tobacco and marijuana use were assessed with frequency questions ranging from “none” to “60+” for alcoholic drinks drunk and cigarettes or marijuana smoked in the last four weeks.

#### Religion

Religious affiliation was included in this study due to the special nature of the sample. Previous studies report associations between religion and SRH [36] with some reviews reporting that this association is unaffected when controlling for demographic variables [37]. Religious affiliation was assessed by asking the participants: “Which of the following best describes your religious belief now?” Options ranged from: 1. “Seventh-day Adventist Christian”, 2. “Other Christian”, 3. “Other Religion”, 4. “No Formal Religion”, and 5. “Don’t Know”. This item was dichotomized to “Non-Adventist” (response 2-4), and “Adventist” (response 1).

#### Social factors

In this study, an Adverse Childhood Experiences score [8–11] was generated by collating responses from the following nine items: 1. “One or both of my parents were in trouble with the law,” 2. “My parents were separated or divorced,” 3. “One or both my parents died,” 4. “One or both parents were absent from home for long periods,” 5. “There were times when family violence occurred,” 6. “There were times when I was physically abused,” 7. “There were times when I was sexually abused,” 8. “One or both parents smoked tobacco,” and 9. “One or both parents drank alcohol weekly or more often.” Each of the nine items included no/yes response options which were given a corresponding value of zero or one. Responses from each item were summed to calculate an overall ACEs score.

Childhood family dynamics were assessed by creating a CFD score from six items, namely: 1. “As a child, my parents showed me love,” 2. “As a child, my parents understood me,” 3. “While I was a child my family had a lot of fun,” 4. “As a child, my parents didn’t trust me,” 5. “As a child, my parents didn’t care what I did,” and 6. “As a child, I enjoyed being at home with my family.” Each item included five response options ranging from “strongly disagree” through to “strongly agree.” Each response was given a corresponding value from one to five and was recoded so that higher scores represented positive outcomes. Responses from each item were summed to calculate the overall CFD score.

### Analysis

The objective of this study was to simultaneously analyze all paths of the hypothesized model (Fig. 2) in order to explore the complexity of the associations between multiple factors and SRH. Hence, structural equation modeling (SEM) [38] was used to estimate the model fit of the data and analyze the direct and indirect effects of the multiple factors in the hypothesized model. Overall model fit was examined using multiple goodness-of-fit indices, namely; chi-square (X^2^) statistic (CMIN), relative X^2^ (CMIN/DF), baseline comparisons fit indices of NFI, RFI, IFI, TLI, CFI, and RMSEA. Structural equation modeling was carried out using AMOS (Versions 24; Amos Development Corporation, Crawfordville, FL, USA). The Bootstrapping method [39] was applied to verify statistical significance of indirect and total effects at *p* < .05. The data were imported into SPSS (version 24; IBM, Armonk, NY) to calculate means, standard deviations, distributions and internal reliability.

## Results

### Descriptive statistics

A summary of descriptive statistics and reliability estimates is shown in Table 1. Sixty-one percent of the students in the study reported “very good” to “excellent” health. This is comparable with the 2014-15 Australian Bureau of Statistics (ABS) survey [40] which reported that 63% of young Australians (aged 15-24 yrs) rated their health as very good or excellent. Unique to the study cohort was that 49% of the students reported an affiliation with a Christian faith and low rates of alcohol consumption (11% reported consuming alcohol in the past four weeks) tobacco use (4% reported using tobacco in the past four weeks) and marijuana use (3% reported using marijuana in the past for weeks).

### The model for factors associated with self-rated health in adolescents

**Fig. 3:**
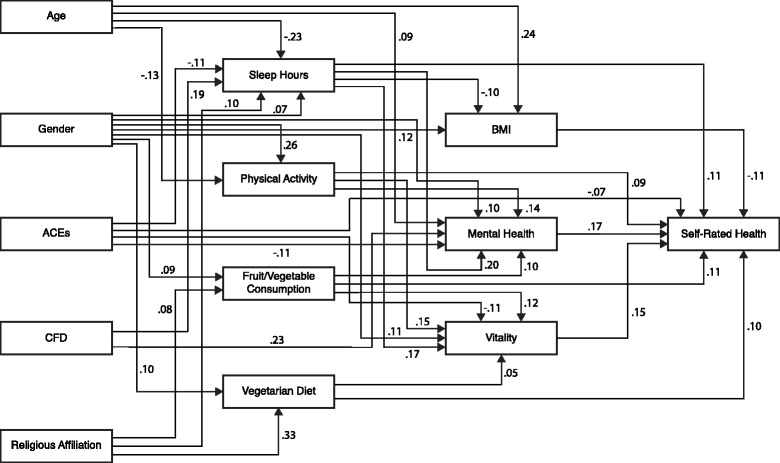
Structural Equation Model Predicting Self-rated Health Status

The hypothesized model (Fig. 2) based on theoretical considerations was submitted for analysis using techniques developed by Jöreskog and Sörbom [41] utilizing an iterative process of inspection of the statistical significance of path coefficients and theoretical relevance of constructs in the model to derive an optimal SEM that best fit the dataset and were theoretically meaningful. The items that asked the participants about alcohol, tobacco, and marijuana use were removed from the model due to their non-significant contributions generating a final structural model (Fig. 3). Modification indices suggested that the health behavior variables be allowed to covary, as well as the health outcome variables mental health and vitality.

The final structural model (Fig. 3) as a whole fitted the data very well, as indicated by the goodness-of-fit indices (CMIN = 33.615; *p* = 0.214; CMIN/DF = 1.201; NFI = 0.976; RFI = 0.933; IFI = 0.996; TLI = 0.988; CFI = 0.996 and RMSEA = 0.016). CMIN/DF statistic below three is considered good model fit [42] as are baseline comparisons fit indices above 0.9 [43]. The RMSEA value was less than 0.06, which indicated a close fit between the data and the model [44]. In Fig. 3, the standardized path coefficients are presented as single-headed arrows, and all shown paths are statistically significant including all indirect and total effect pathways.

The final structural model (Fig. 3) describes the upstream associations of BMI, mental health and vitality, health behaviors, demographics and social factors on SRH as well as their interactions. The squared multiple correlation calculated for SRH was 0.20 which indicates that the model explained 20% of the variance in self-rated health.

Based on standardized path weight coefficients (β’s), the health outcome variables BMI (β = − 0.11), mental health (β = 0.17) and vitality (β = 0.15) had a direct association with SRH. This indicates that adolescents who reported a higher BMI reported a poorer SRH, and adolescents who reported higher mental health and vitality scores reported better SRH.

The health behavior variables sleep hours (β = 0.11), physical activity (β = 0.09), fruit/vegetable consumption (β = 0.11) and a vegetarian diet (β = 0.10) had a direct association with SRH. This indicates that adolescents reporting more sleep each night, more physical activity, greater consumption of fruit and vegetables and a vegetarian diet also reported a better SRH. The health behavior variables were also associated with SRH indirectly through the health outcome mediating variables. Sleep hours was associated with SRH indirectly through the mediating health outcome variables BMI, mental health and vitality. Physical activity was associated with SRH indirectly through the mediating health outcome variables mental health and vitality. Fruit/vegetable consumption was associated with SRH indirectly through the mediating health outcome variables mental health and vitality. A vegetarian diet was associated with SRH indirectly through the mediating health outcome variable vitality. Of the health behavior variables, sleep hours had the strongest combined direct and indirect association with SRH (β_total_ = 0.178) followed by fruit/vegetable consumption (β_total_ = 0.144), physical activity (β_total_ = 0.135) and then vegetarian diet (β_total_ = 0.103).

Of the demographic and social variables, ACEs was the only variable that had a direct association with SRH (β = − 0.07) with the other demographic and social variables indirectly associated with SRH. Age was associated with SHR through the mediating health behavior variables sleep hours and physical activity, and through the mediating health outcome variables BMI and mental health. Gender was associated with SHR through the mediating health behavior variables sleep hours, physical activity, fruit/vegetable consumption, and vegetarian diet, and through the mediating health outcome variables BMI, mental health, and vitality. ACEs was associated with SHR directly and through the mediating health behavior variable sleep hours and the mediating health outcome variable mental health and vitality. CFD was associated with SHR through the mediating health behavior variable sleep hours and through the health outcome variable mental health. Religious affiliation was associated with SHR through the mediating health behavior variables sleep hours, fruit/vegetable consumption and vegetarian diet.

Notably, of the demographic and social variables in the model, ACEs had the strongest association with SRH (β_total_ = − 0.125). Hence, more ACEs were associated with lower SRH. Gender had the second strongest association with SRH of the demographic and social factors (β_total_ = 0.092) and also interacted with the greatest number of the mediating variables in the model. The association of age on SRH (β_total_ = − 0.067) demonstrated that older adolescents reported poorer SRH, however, overall, males rated their health better which is in line with other studies [13]. The association of CFD (β_total_ = 0.047) on SRH demonstrated that adolescents reporting better CFD also reported better SRH. Finally, the model indicated that although the respondent’s religion did have indirect links to SRH its association was small (β_total_ = .005). Adolescents who identified as Adventist were more likely to report higher SRH, and better health behaviors than those who identified themselves as not affiliated with the Adventist Church.

## Discussion

This study explored concomitantly the relationships between factors associated with SRH in adolescents attending Adventist schools in Australia. By including a number of variables into one conceptual model and analyzing them simultaneously, the study is unique in that it was able to describe a complex network of associations between the factors that influence SRH. This study supports the need for a broad multi-component approach to the study of adolescent health.

The findings in this study demonstrate the association between mental health and SRH which is in line with findings from previous studies [20, 22]. The mental health measure used in this study had the strongest association with SRH of the three health outcome variables measured and was associated with the most health behaviors, demographics and social variables in the model. Several health behaviors (sleep hours, physical activity, and fruit/vegetable consumption), as well as demographics (age and gender), and social factors (ACEs and CFD) had a direct association with mental health. Notably, the association between the adolescent’s childhood upbringing (ACEs and CFD) and mental health demonstrates how social factors early in life are associated with mental health status years later in adolescence.

The vitality metric used in this study (a measure of energy and fatigue status) had the second strongest association with SRH of the health outcome variables. All health behaviors in the model (sleep hours, physical activity, fruit/vegetable consumption, vegetarian diet) along with gender and ACEs directly associated with the measure vitality. Research on vitality is limited; however, one study found that up to 30% of healthy teens experience symptoms of fatigue that affect their normal functioning [45]. The observed influence of health behaviors on vitality in this study highlights the importance of targeting healthy behaviors for improving the energy levels and lessening fatigue among adolescents.

There is a wealth of literature supporting the importance of health behaviors on adolescent health [3], however, a unique aspect of this study was the simultaneous assessment of the association of four health behaviors (sleep hours, physical activity, fruit/vegetable consumption, vegetarian diet) with SRH. This allowed the health behaviors to be ranked according to their strength of association with SHR. While all health behaviors had a direct association with SRH and an indirect association through one or more of the health outcome variables, sleep had the strongest association with SRH, followed by fruit/vegetable consumption, physical activity, and vegetarian diet. This finding highlights the value of prioritizing healthy sleep hygiene among adolescent cohorts [46], although clearly, interventions that address all health behaviors are likely to be most efficacious and therefore desirable.

In the SEM analysis, the items measuring the health behaviors: consumption of alcohol and the use of tobacco and marijuana had non-significant pathways to SRH. It is well documented [5] that these health behaviors influence adolescent health negatively. A possible explanation for the non-significant effect of these health behaviors in this study may be that the study cohort reported a low prevalence of these behaviors. While this low prevalence was expected given the Adventist community proscribes such behavior, further exploration as to what motivates the use of alcohol, tobacco and marijuana in a low using cohort would be of interest.

Of the selected demographics and social factors included in the model predicting SRH, ACEs presented as having the strongest association. Indeed, it is remarkable that adolescents who reported higher incidents of adverse experiences in their earlier childhood, reported poorer SRH in their adolescent years. Although children may have no choice in the ACEs they experience, this study reinforces the necessity for childhood human rights, health promotion and resilience building [47] to be at the forefront of global policy and intervention development to provide benefits not only in childhood, but also later in adolescent life.

Of the five demographic and social factors assessed, gender had the second strongest association with SRH, and was associated with the most number mediating variables, interacting with all health behavior and health outcome variables in the model. This suggest that interventions targeting improving general health of adolescents may be more effective if they were gender specific. The influence of CFD and religion on SRH in this model is noted, albeit not as strong as ACEs and gender.

### Strengths and limitations

The strength of this study is that it concomitantly explored a number of factors associated with SRH and describe the complex interaction between these factors and SRH. It is acknowledged, however, that model presented in this study, although strong, represents only part of the big picture of the overall influences of SRH. For example, socio-economic status is a well-known predictor of SRH [17], and this was not assessed in this study as no data on socioeconomic status was collected. Another limitation of this study is that it focused on a comparatively homogeneous group of adolescents who were exposed to a faith-based community, namely, Adventist Christians who place a strong emphasis on health and a wholistic lifestyle. Since its inception in 1863, the Adventist religion has promoted the adoption of a healthy lifestyle to its members that includes regular exercise, a vegetarian diet and rest. Alcohol, caffeine, tobacco and illicit substances are also proscribed (Fraser, 2003). The Adventist population has been the focus of numerous health studies as they tend to experience good health and lower rates of disease [48]. Adventist schools espouse the health practices of the Adventist church. Hence, while approximately half of this study cohort did not identify themselves as Adventist, they were likely influenced the by health focus of the Adventist church. It is possible that the adolescents in this study potentially underscored their self-rated health status compared to adolescents in the general population due to the high health ideals advocated by the faith-based schools they attend. This may have resulted in these adolescents perceiving and judging “very good” or “excellent” health against a more rigorous standard. This limits the generalization of the findings to other populations. The cross-sectional nature of this study means that only associations could be measured, it is not possible to say whether these relationships were causal. Although SRH has been established as a legitimate and stable construct for use in adolescent populations [13–21] to measure general health status, objective measures of health including biomedical testing as represented in the conceptual framework for determinants of health [12] may improve the validity of the findings in this study.

## Conclusion

This study presented a conceptual model that described the complex network of factors concomitantly associated with SRH in adolescents. The results highlight the association of mental health with SHR. Gender-sensitive interventions prioritizing modifiable health behaviors such as sleep, healthy diet, and physical activity may achieve a greater combined effect in improving adolescent health status than select single factor interventions. The association between ACEs and adolescent SRH reinforces the necessity to address childhood human rights, resilience, family dynamics, and health promotion in children for lasting benefits later in adolescent life. Further research into what influences the variables interacting with SRH may provide insight into more effective interventions to improve adolescent health.

## Abbreviations

ABS: Australian Bureau of Statistics
ACEs: Adverse childhood experiences
BMI: Body mass index
CFD: Childhood family dynamics
CFI: Comparative Fit Index
CMIN: Chi-square statistic
CMIN/DF: Relative X^2^
IFI: Incremental fit index
NFI: Normed Fit Index
RFI: Relative fit index
RMSEA: Root Mean Square Error of Approximation
SEM: Structural equation modeling
SRH: Self-rated health
TLI: Tucker Lewis index
X^2^: Chi-square
